# What We Know About per- and Polyfluoroalkyl Contamination Levels in Milk. A Review from the Last Decade

**DOI:** 10.3390/foods14132274

**Published:** 2025-06-26

**Authors:** Dalia Curci, Tamil Selvi Sundaram, Sergio Ghidini, Francesco Arioli

**Affiliations:** Department of Veterinary Medicine and Animal Science, University of Milan, Via dell’Università 6, 26900 Lodi, Italy; dalia.curci@unimi.it (D.C.); tamil.sundaram@unimi.it (T.S.S.); sergio.ghidini@unimi.it (S.G.)

**Keywords:** milk, PFAS, mass spectrometry, review

## Abstract

Due to its nutritional value and versatility, milk is recognized to be one of the most widely consumed beverages worldwide, with a forecasted global consumption of 356 million tons in 2025. Recent updates in legislation and the assessment of their toxicological effects increased attention to the determination of per- and polyfluoroalkyl substances (PFASs), classified as “forever pollutants” for their stability and environmental spread in foods. The present review aims to collect all the information related to milk contamination reported by the scientific literature over the last decade and address the PFAS contamination levels around the globe. Twenty-two studies were found to be published about the detection of PFASs in milk in the period considered in this review, with a total of 824 analyzed samples on a global scale and a maximum of 60 investigated analytes. This review confirms that PFASs, including PFOA and PFOS, are detected in milk worldwide. However, the wide variability in reported concentration levels highlights a critical lack of consistent data and underscores the absence of coordinated, large-scale monitoring programs. This gap in surveillance significantly limits our understanding of actual exposure risks and reinforces the urgent need for comprehensive, systematic research across different regions and production systems.

## 1. Introduction

Among the most widely consumed beverages worldwide, dairy milk is recognized to be one of the most important ones, both for its nutritional value and versatility. In fact, for centuries, milk has been integral to human diets not only because it is rich in essential nutrients (e.g., calcium, proteins, vitamins, and minerals) but also for its role as a base for a variety of dairy products such as cheese, yogurt, and butter, playing a key role in the diets of millions, especially for infants and young children [1]. Trends in global milk consumption show stable growth from 2019 and forecast a global consumption of 356 million tons of milk in 2025 [2].

As the global demand for milk and dairy products continues to increase, concerns about milk contamination and related potential human risks have emerged as significant health issues. Contaminants can be present in milk and dairy products through multiple pathways, including feed, water sources, and dairy cows [3,4].

Due to recent updates in legislation and the assessment of their toxicological effects, great attention has been paid to the determination of per- and polyfluoroalkyl substances (PFASs) [3]. PFASs are classified as “forever pollutants” because of their stability and widespread environmental impact. Moreover, these contaminants are known for their ability to move across the environment and accumulate throughout the food chain [5]. While PFAS can cycle through water, soil, and food chains, affecting human health via various exposure routes, diet and drinking water remain the primary sources of human PFAS exposure [6,7]. Estimates show that 60–98% of the total PFAS daily exposure for humans can be linked to dietary contribution [8], and the European Food Safety Authority (EFSA) has designated milk and dairy products as significant sources of PFAS chronic exposure for sensitive populations [9].

Diet exposure may occur through both direct environmental contaminations, affecting raw and non-processed foods, and indirect exposure from PFAS present in food contact materials, a pathway that remains controversial [10]. Concerns are linked to PFAS’s ability to act as endocrine disruptors, and to affect the human immune, endocrine, and reproductive systems [7]. Moreover, there is increasing concern on the topic since the “International Agency for Research on Cancer” (IARC) categorized perfluorooctanoic acid (PFOA) as “carcinogenic to humans” (Group 1), and perfluorooctanesulfonic acid (PFOS) as “possibly carcinogenic to humans” (Group 2B) [11].

Several approaches have been used worldwide to limit and contain the damage caused by PFAS, including restricting or eliminating their use in common materials, and allocating funds for remediation efforts [12]. At the European level, authorities began monitoring PFAS contamination in 2004, and in 2008, the European Union (EU) introduced regulations on PFOS production and commercialization, valid until 2019, which was then replaced by the “Regulation (EU) 2019/1021,” which prohibits the manufacturing and sale of PFOS and its derivatives (with some exceptions) [13]. Moreover, in 2008, EFSA issued a scientific opinion on PFOS and PFOA, acknowledging that the contribution of PFAS precursors to environmental contamination was not fully understood [14]. In 2018, EFSA assessed human health risks from PFOS and PFOA in food, noting widespread exposure in blood samples and emphasizing the need for new analytical methods and legislative measures [15]. In 2020, the EFSA Panel on Contaminants in the Food Chain (CONTAM) published a scientific opinion assessing the health risks of four PFAS compounds (PFOS, PFOA, perfluorononanoic acid (PFNA), and perfluorohexanesulfonic acid (PFHxS)) in food, establishing a tolerable weekly intake (TWI) for their sum of 4.4 ng per kg of body weight per week [16]. Consequently, the EU Commission established maximum levels (ML) for these four PFASs (singularly and as a sum) in certain foods [17]. Despite all the legislation mentioned before, there are still no limits set for PFASs in milk and dairy products, but the Commission Recommendation (EU) 2022/1431 established guideline values [18].

In scientific literature, studies [19,20,21,22] have demonstrated that PFASs are capable of binding to β-lactoglobulin proteins in milk, and consequently that dairy milk can become a reservoir for PFAS. This underlines the importance of better understanding how PFAS can accumulate in milk and dairy products (transfer of PFAS from feed to cows, production process, packaging) [4], and collecting the available data on the global contamination levels to provide useful information that might support PFAS regulation in this matrix.

The present review aims to provide a comprehensive and up-to-date overview of the topic and collect most of the information related to milk PFAS contamination reported by the scientific literature over the last decade, considering (i) the main methodology and investigated analytes; (ii) the number of samples analyzed on a global scale; and (iii) PFAS contamination levels of milk around the globe. Moreover, this review could provide useful insight for those who want to evaluate a risk characterization for humans based on milk consumption, addressing and considering the most recent data available in scientific literature. This review focuses on literature from the past ten years to ensure relevance, accuracy, and alignment with current scientific and regulatory standards. Advances in analytical technologies over the last decade have significantly improved the sensitivity and specificity of PFAS detection in complex matrices like milk. Furthermore, recent studies reflect updated regulatory priorities, including monitoring both legacy and emerging PFAS compounds. Limiting the scope to the most recent decade also accounts for changes in contamination sources and industrial practices. Additionally, contemporary sample preparation techniques enhance comparability and data quality across studies. Finally, this time frame provides a manageable and focused dataset, allowing for a clearer synthesis of current knowledge and identification of research gaps in PFAS detection across different milk types.

## 2. Materials and Methods

This review selected publications based on scientific studies, considering ten years (2014–2024), and the first trimester of 2025, and excluding both non-peer-reviewed studies and studies not available in English. The literature search was performed by using bibliographic databases, such as PubMed, Scopus, and Google Scholar. The different keywords used were PFASs, milk, PFASs “and” milk, PFASs “and” dairy, PFASs “and” goat, PFASs “and” sheep. To enrich the information, we included in this review studies with a reduced number of samples. The studies that did not report the number of milk samples analyzed were cited but excluded from the discussion of this review. The initial search for studies on PFAS in milk yielded 117 results in PubMed and 164 in Scopus. A broader search conducted using Google Scholar returned approximately 24,800 results. However, the majority of these were found to be irrelevant to the specific focus of this review and were rapidly screened out during the initial assessment phase. The authors performed the research on each database and screened each work independently. The selected studies were then discussed in pairs. Applying our inclusion criteria, excluding the duplicates, 22 studies were selected for this review.

We acknowledge that restricting the research to only original articles (published in English) and not considering also institutional reports might lead us to underestimate the number of samples. Nevertheless, we are confident that none of these methodological limitations would change the overall conclusions of this review.

Abbreviations of the analytes cited in this review are reported in the Abbreviations section at the end of the document.

## 3. Results

### 3.1. Literature Overview

**Figure 1:**
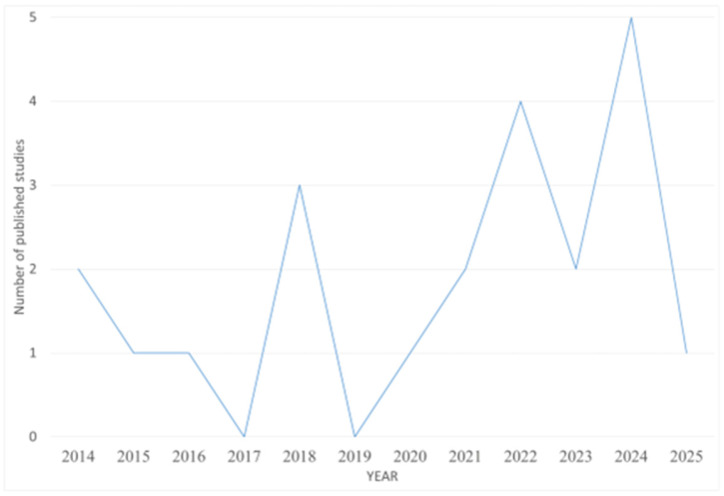
Line chart reporting the number of studies published each year from 2014 to 2025 related to the detection of PFASs in milk samples.

**Figure 2:**
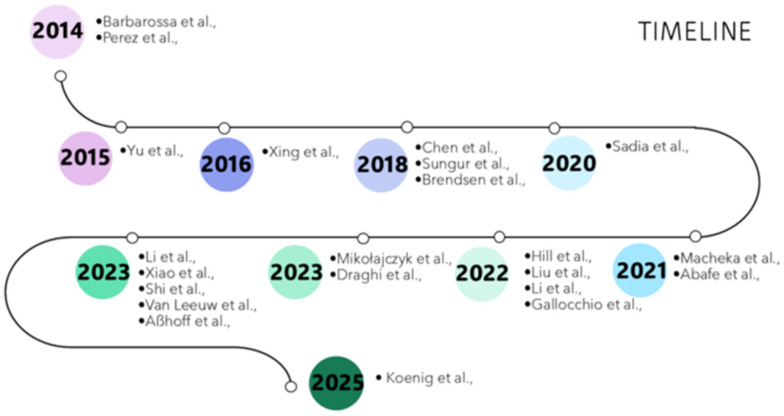
Timeline showing the different studies published around the world during the 2014–2025 decade related to the detection of PFASs in milk samples [4,8,23,24,25,26,27,28,29,30,31,32,33,34,35,36,37,38,39,40,41,42].

Considering our selection criteria, 22 studies were found to be published about the detection of PFASs in milk in the period considered in this review (Figure 1 and Figure 2). Detailed information is reported in Table 1.

Current methods for detecting PFASs in milk rely on HPLC-MS/MS. Due to the amphipathic PFAS characteristics, they can accumulate in both protein and fat; therefore, precise sample preparation is crucial to accurately detect and quantify these analytes [31]. Table 1 shows that different PFAS extraction techniques have been explored. The extraction protocol usually involves two steps: firstly, a liquid extraction; and secondly, a clean-up step performed by SPE. However, some studies performed the extraction and purification in one step by using a QuEChERS method.

Considering the investigated analytes (Table 2), while all the studies involved PFOA and PFOS, five of them [23,26,28,29,30] did not consider all the four main PFASs (PFOA, PFOS, PFNA, PFHxS) (Table 1). Perfluorocarboxylic acids and perfluoroalkyl sulfonic acids were included in all the studies; however, only a few of them considered fluorotelomers, which can be industrially used as an alternative to PFOA and PFOS. The highest number of analytes investigated was by Li et al. 2024 [37], who developed a method able to discriminate between 60 PFASs in milk.

### 3.2. Incidence of PFASs in Milk Around the World

**Figure 3:**
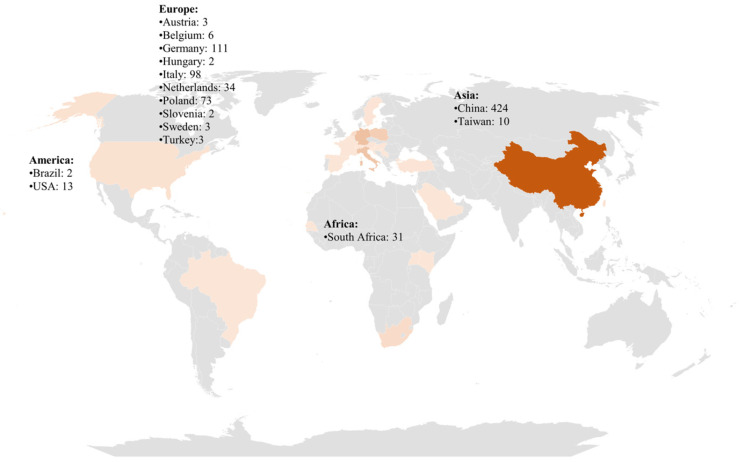
The number of milk samples analyzed in different countries for the detection of PFASs. All the countries with more than one sample analyzed are listed as text, divided by the continent. Darker shades of orange indicate countries with a higher number of samples.

**Figure 4:**
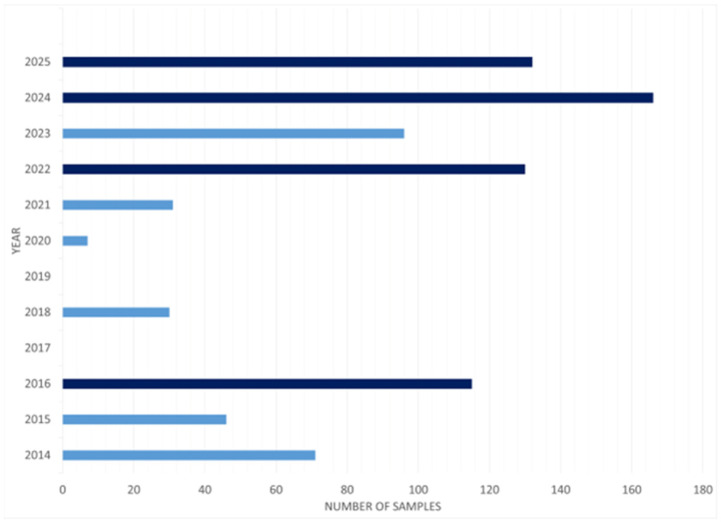
Number of milk samples analyzed each year from 2014 to 2025 for the detection of PFASs. In darker blue the years in which the number of analyzed samples was higher than 100.

A total of 824 milk samples were analyzed in the period between 2014 and 2025 (Figure 3 and Figure 4), also including 22 goat and 15 sheep milk samples. In the following paragraphs, we summarize the main findings of the 22 selected studies divided by the origin of the samples collected. Moreover, in Table S1, we collected all the information provided by the several considered studies regarding the concentration of the four main PFASs (PFOA, PFOS, PFNA, PFHxS). In this table, provided in the Supplementary Materials, we reported the results as they are shown in the original article, without changing the measurement units.

#### 3.2.1. Africa

Two studies published in 2021 report the detection of PFASs in milk and infant formula available on the South African market. In particular, Macheka et al. 2021 [8] analyzed the presence of 15 PFASs in 23 dairy milk samples distributed as follows: five fat-free, eight low-fat, seven full cream, and three lactose-free. Their findings showed that the sum of the 15 analyzed PFAS in the dairy milk ranged from 0.08–15.51 ng mL^−1^ with PFBA, PFPeA, PFuDA, PFTrDA, and PFDoA being the most prevalent PFASs and a detection frequency of ≥96%. Despite the decrease in the PFAS concentrations from full cream to low fat, no statistically significant difference was reported for the different types of milk.

Moreover, Abafe et al. 2021 [31] developed a method to assess the presence of 15 PFASs in milk and infant formula, applying it to eight pooled dairy milk samples. The analytes detected more frequently in retail milk were PFDA, PFuDA, PFDoA and PFTrDA. This study reported that the presence of perfluoro carboxylic acids was prevalent compared to perfluoroalkylsulfonic acids. Detailed information related to the concentration of the four main PFASs is reported in Table S1 provided in the Supplementary Materials.

#### 3.2.2. America

Hill et al. 2022 [4] improved a sensitive method to detect 27 PFASs in milk and applied it to 13 milk samples collected from six states across the United States. In particular, samples were collected from dairy farms that confirmed the use of biosolids on cropland or were within geographic proximity to military installations with confirmed aqueous film-forming foam (AFFF) use. The only analyte detected in this study was 6:2 FTS at a concentration of 6.6 ng L^−1^, while no other PFASs were detected in the study. Detailed information related to the concentration of the four main PFASs is reported in Table S1 provided in the Supplementary Materials. According to the USA, the Food and Drug Administration (FDA) has been testing food for the detection of PFASs since 2019, with most of them collected for the total diet study. Eight sets of data have been published since then, and three milk samples have been analyzed throughout these years in each dataset. The results were always below the method detection limits [43].

#### 3.2.3. Asia

Eight studies have been published over the last decade on the detection of PFASs in milk samples collected in Asia. Firstly, Yu et al. 2015 [25] analyzed 46 cow milk samples from different brands purchased in sampling sites in China in the provinces of Shandong, Anhui, Liaoning, Fujian, Jiangxi, and Guangdong. In total, 16 out of the 20 investigated PFASs were found in the samples. PFBA, PFNA, PFHxDA, and PFTrDA were the most frequently detected compounds (89% of the milk samples), while the total concentration of PFASs ranged from 0.19 to 0.66 μg L^−1^.

Xing et al. 2016 [26] assessed the presence of PFOS and PFOA in milk by analyzing 115 samples, divided as follows: 24 raw milk and 91 retail milk from 8 different producing areas in the Chinese autonomous region of Xinjiang. Both the analytes were found in retail samples, while in raw milk PFOA was not detected. The percentage of the quantifiable samples was 33% and 39.6% for PFOA and PFOS in retail milk, respectively, while it was 12.5% for PFOS in raw milk. The mean concentrations detected were 16.2 and 24.5 ng g^−1^ for PFOA and PFOS in retail milk, respectively, while it was 2.2 ng g^−1^ for PFOS in raw milk. In this study, no correlation was found between the measured concentrations of the two compounds in each sample.

Chen et al. 2018 [27] detected and quantified 7 PFASs in different food items, including 10 milk samples, purchased from Taiwan. According to the results, the median concentration levels were 0.01, 0.03, 1.70, 1.54, 0.02, and 0.07 ng mL^−1^ for PFHxS, PFHxA, PFOA, PFDA, PFUnDA, PFDoDA, respectively, while PFOS was not detected. The number of milk samples analyzed by Li et al. 2022 [33] was four (two raw milk, two bagged milk), with all being obtained from local farms and markets. In this study, the most detected compound was PFOS, while PFPeA was detected in only one bagged sample.

Liu et al. 2022 [34] investigated the occurrence of 16 PFASs in 107 raw milk samples purchased from nine Chinese provinces, investigating potential correlations between PFAS concentrations in milk and cow feed. The study showed a detection frequency of 96.3% for PFASs in milk samples with PFOS (67.5%) and PFBA (12.8%) as prevalent compounds, with sum concentrations of the analyzed PFASs ranging from below the method detection limit to a maximum of 9.82 ng g^−1^. The highest detected concentration is related to PFOS (9.19 ng g^−1^).

Li et al. 2024 [37] developed a method to determine 60 PFASs in milk purchased in Beijing, China and applied it to 35 milk samples. Among all the investigated PFASs, only PFBS, PFHxS, PFOS, PFNS, PFPeA, PFOA, PFNA, and 6:2 FTS were detected.

Xiao et al. 2024 [38] investigated the occurrence of PFAS in cow feed, drinking water, and raw milk, exploring the passage of PFAS through these matrices. In this study, 92 raw milk samples were analyzed from 20 provinces across China. Seven PFAS were detected in raw milk with a mean concentration of ∑PFAS of 0.15 ± 0.13 ng mL^−1^, with the highest detection frequency for PFOS and PFOA. In particular, PFOA (0.08 ± 0.09 ng mL^−1^) was the most significant compound in raw milk, contributing to 51.5%.

Shi et al. 2024 [40] developed a method to detect 22 PFASs in milk and applied it to analyze 25 milk samples (whole milk, low-fat milk, skim milk, and fresh milk). The detected PFASs were PFBA and PFOA, with detection frequencies of 67% and 10%, respectively. Detailed information related to the concentration of the four main PFASs is reported in Table S1 provided in the Supplementary Materials.

#### 3.2.4. Europe

The first study published on the topic, considering ten years from 2014 to 2025, is by Barbarossa et al. 2014 [23], who performed the first monitoring study on the presence of PFOS and PFOA in cow milk available in the northern Italian market on a large scale. This study investigated 67 samples: 22 samples obtained from different stores in the Bologna areas, and 45 samples (8 organic milk; 37 from farms with high hygienic and nutritional standards) represented all producers supplying milk to two dairy plants in Lombardy and Emilia Romagna. According to the findings, PFOS was present in 43% of the samples, with a maximum concentration of 97 ng L^−1^, while PFOA was present in 40% of the samples, with a maximum concentration of 32 ng L^−1^. No correlation was found between the type of milk and the detected levels of contamination.

Sungur et al. 2018 [28] investigated the presence of PFOA and PFOS in Turkish brands of food and beverages available on the Turkish market, including three milk samples, detecting an average PFOS concentration of 0.100 ng g^−1^, while PFOA was not detected. Berendsen et al. 2018 [29] presented a study in which they developed a sensitive method for 13 PFASs in milk, applying it to 17 milk samples (16 cattle, 1 goat) purchased from the Netherlands. The real case application was focused on the detection of PFOA and HFPO-DA only, which were not found in the samples. Gallocchio et al. 2022 [32] developed a fast method to determine 14 PFASs in food of animal origin, including cow’s milk, and applied it to 6 milk samples. No PFASs were found (<LOD).

Mikołajczyk et al. 2023 [35] analysed 73 milk samples to determine the presence and concentration of 14 PFASs. Samples were distributed as follows: 38 cow, 20 goat, and 15 sheep milk samples, all gathered from several regions of Poland. Results showed that the lower-bound concentration sum of the four regulated PFAS (∑4 PFASs: PFOA, PFOS, PFNA, PFHxS) were 0.0055, 0.0046, and 0.0008 µg kg^−1^ in sheep’s, goat’s, and in cow’s milk, respectively. Among them, linear PFOS was the most detected, with detection percentages of 33%, 76%, and 93% of cow’s, goat’s, and sheep’s milk samples, respectively. Considering the other investigated PFASs, the predominant was PFTeDA, the highest concentration of all 14 investigated PFASs, peaking in sheep’s milk at a mean of 0.0073 µg kg^−1^.

Draghi et al. 2023 [36] evaluated the distribution of 14 PFASs in the 3 milk fractions (whole milk, skim milk, and cream), collecting the samples from 23 cows in northern Italy. In this study, PFBA was shown to be the most frequent PFAS detected in all three fractions, followed by PFOS and PFOA. According to the four regulated PFASs, the highest detected concentration was related to PFOA in the skimmed fraction (1002.70 pg g^−1^), while PFHxS was detected only in the cream fraction. Moreover, they found a significant difference in PFOS concentrations, which was significantly higher in milk cream than in skimmed and whole milk. Ten milk samples, collected from the German market, were analyzed by Aßhoff et al. 2024 [41]; in this study, PFOS was detected in seven samples, but only quantifiable in three samples of them (concentration range of 9.0–13 ng L^−1^). PFHxA was also detected in one sample.

Van Leeuw et al. 2024 [39] analyzed 17 milk and milk products, including 4 milk samples, to investigate the presence of 25 PFASs collected from the Belgian market. In this case, all the investigated analytes were always detected below the LOQ. The first study published on this topic in the first trimester of 2025 was by Koenig et al. [42] who analyzed 132 milk samples collected from the German market. According to the samples provenance they were distributed as follows: 2 Hungary, 2 Slovenia, 1 Switzerland, 3 Austria, 17 The Netherlands, 1 Croatia, 2 Italy, 1 France, 2 Belgium, and 101 Germany. Considering the four main PFASs, the detected percentages were 98.4%, 96.2%, 2.27%, and 47%, for PFHxS, PFOS, PFOA, and PFNA, respectively. These analytes were detected with a median concentration sum of 4.57 ng kg^−1^ (range 0.941–137 ng kg^−1^). This study included also one goat’s milk sample, which showed a maximum concentration of 137 ng kg^−1^ for the four main PFASs (PFOA, PFOS, PFNA, PFHxS). Detailed information related to the concentration of the four main PFASs is reported in Table S1 provided in the Supplementary Materials.

#### 3.2.5. Others

Two studies assessed the presence of PFASs in milk samples collected from different nations.

Pérez et al. 2014 [24] assessed the presence of 21 PFASs in several food items taken from different countries including Brazil, Saudi Arabia, Spain, and Serbia. Among these items, they analyzed four milk samples (one concentrated milk sample from Brazil, one whole milk from Saudi Arabia, one sterilized and one fresh milk from Serbia). In this study, they reported the summary of the concentrations of target compounds (pg g^−1^) in positive samples but did not show the specific food item. As a general conclusion, it is possible to note that, according to their lipophilicity, PFOA was mainly found in skimmed milk, whereas PFOS was present in whole milk.

Sadia et al. 2020 [30] developed a method for the detection of five PFASs (PFOS was analyzed as l- and br-PFOS) in different food items and applied it to seven milk samples from different countries (Sweden, Kenya, Senegal, Uganda, and Brazil). The highest concentration was found for PFOS in a sample from Kenya (89.9 pg g^−1^). However, due to the small sample size, the detected concentrations might be considered indicative, but not representative of the national average concentration.

Detailed information related to the concentration of the four main PFASs is reported in Table S1 provided in the Supplementary Materials.

## 4. Discussion

### 4.1. Understudied Exposure: PFAS in Milk Amid Widespread Consumption

Milk—whether from cows, goats, or less conventional sources—has long been a cornerstone of human nutrition, yet its role as a potential vector for environmental contaminants like PFAS calls for closer and more comprehensive examination. A recent review has substantially deepened our understanding of PFAS contamination in human breast milk [44], highlighting early-life exposure as a critical concern. However, while the focus on breast milk is undoubtedly important, it primarily pertains to a relatively short—but fundamental—period, typically the first year of life. In contrast, as shown in this review, far less attention has been given to PFAS contamination in other types of milk, particularly cow milk, which is culturally and nutritionally significant across all age groups and commonly consumed throughout life [1]. This disparity in research focus is noteworthy, especially considering that cow milk forms a major component of the human diet in many regions and supports a vast and diverse market, encompassing both small-scale local producers and large industrial dairy operations. The limited data available on PFAS levels in the several milk types available in the market poses a challenge for comprehensive dietary exposure assessments and hinders the development of effective regulatory measures. These findings underscore the urgent need for further investigation into PFAS contamination in dairy products beyond breast milk to ensure food safety and protect public health across all life stages.

To the author’s knowledge, this is the first review to provide both comprehensive information about global data on PFAS concentrations in milk and a dataset that could facilitate rapid data evaluation—particularly for applications such as risk assessment.

A report from FAO projected that “consumption of milk and dairy products will rise from 45 kg year^−1^ per capita to 66 kg in developing countries, and from 212 to 221 kg in industrial [45]. In particular, milk consumption patterns exhibit substantial regional variation, influenced by cultural, economic, and demographic factors. In sub-Saharan Africa, per capita milk consumption has remained largely stagnant over the past three decades. Milk’s contribution to dietary energy and protein intake has remained constant, suggesting that increases in milk availability have merely kept pace with population growth. As a result, only minor increases in consumption are projected in the coming years. In the Near East and North Africa, milk—alongside poultry—constitutes a significant share of animal product consumption. The contribution of animal products to total caloric intake is expected to rise modestly, from 8.7% in 1997/99 to 11.4% by 2030, reflecting a gradual dietary transition. In Brazil, milk consumption is notable for its upward trajectory. As of 1997/99, per capita consumption was slightly over half the level observed in industrialized nations. This figure is projected to reach approximately three-quarters of the industrial country average by 2030, highlighting Brazil’s growing reliance on dairy as a dietary staple. By contrast, in the broader Latin America and Caribbean region (excluding Brazil), dietary patterns are more heavily influenced by meat, with no significant emphasis on milk consumption reported. South Asia, excluding India, demonstrates a different trend, with milk already playing a central role in the diet. Per capita milk consumption is 50% higher than the average for developing countries and continues to grow steadily. This reflects a culturally embedded preference for dairy products in the region. In India, milk and milk products are the primary drivers behind the projected increase in animal product consumption. The importance of dairy is expected to continue growing until 2030, further cementing its role in the Indian diet. In East Asia (excluding China), the increase in animal product intake is largely due to rising meat consumption, and milk does not appear to contribute significantly to dietary changes. Similarly, in China, milk consumption is projected to remain low, increasing only from 7 kg per capita per year in 1997/99 to 14 kg by 2030. Dairy products will continue to play a minimal role in the Chinese diet, in contrast to the country’s rapidly growing consumption of pork, poultry, and eggs [45].

Thus, data from the Food and Agriculture Organization (FAO) reveals that the countries with the highest per capita cow milk consumption are primarily located in Europe, Oceania, and parts of Asia. In particular, considering Europe, the countries with the highest per capita cow milk consumption are Sweden, Netherlands, Denmark, Ireland, and Germany, with milk consumption ranging from 350 to 270 kg year^−1^ per capita [45]. These findings underscore the heterogeneity of milk consumption globally, with significant implications for nutrition policy, agricultural development, and food system sustainability.

When comparing the geographic distribution of PFAS studies in milk with global milk consumption patterns, a notable imbalance emerges. Countries with high per capita cow milk consumption—such as Sweden, the Netherlands, Denmark, Ireland, and Germany—have contributed relatively few studies to the literature, with only Germany and Sweden appearing in recent investigations. Even within these, sample sizes are often limited, and coverage is not representative of the national level. This is particularly striking given that per capita consumption in these countries ranges from 270 to 350 kg per year, placing them among the world’s top consumers. Similarly, in rapidly growing dairy markets such as Brazil and India—where milk plays a central or increasingly significant dietary role—there is a marked absence of published data on PFAS contamination in milk, despite projections indicating major increases in consumption by 2030. Conversely, some countries with low or stagnant milk consumption, such as China and South Africa, have been the focus of multiple PFAS studies. While these studies provide important insight into regional contamination and exposure, the overall evidence base remains geographically skewed and insufficiently aligned with actual consumption trends. This discrepancy underscores the urgent need for targeted PFAS monitoring in countries with high or rising milk consumption, to ensure accurate exposure assessments and inform relevant regulatory frameworks.

Thus, it is essential to critically assess the available data—considering both the number of samples and their geographic origin—while also emphasizing the significant lack of information from several countries, which limits the global representativeness of current findings, which is a key finding of this review.

### 4.2. PFAS Contamination: Regional and Analytical Disparities

Exploring the results shown in the previous section, the comparative analysis of PFAS concentrations in milk across continents reveals notable geographic disparities in both contamination levels and compound diversity. Asia exhibits the broadest spectrum of PFAS types, with studies reporting the detection of up to 16 different compounds in milk samples, including PFBA, PFNA, PFTrDA, and PFOS, with concentrations ranging from below detection limits to 9.82 ng g^−1^. In contrast, while displaying less compound diversity, Europe shows relatively higher concentrations of specific PFASs, particularly PFOS and PFOA. Notably, PFOS was found at 97 ng L^−1^ concentrations in Italy [23] and up to 137 ng kg^−1^ in Germany [42]. Africa and America reported generally lower PFAS concentrations, with a few exceptions such as the detection of 15.51 ng mL^−1^ in South African dairy milk [8], while U.S. samples consistently showed concentrations below detection limits, except for one sample with 6:2 FTS at 6.6 ng L^−1^ [4]. The FDA Total Diet Study also supports the minimal PFAS contamination in U.S. milk. This contrast underscores a trend: Asian samples tend to reflect environmental and agricultural variability through a wider PFAS profile, while European studies highlight localized high concentrations of fewer, often regulated PFASs, such as PFOS and PFOA. These findings suggest differing sources, regulations, and environmental persistence of PFASs across regions.

Considering the large number of compounds included in the PFASs category, and the need to collect comparable data from different studies, this review highlighted that it would be interesting and useful to (i) expand the range of investigated molecules in future studies; (ii) define a list of required principal molecules to be included in the study. As emerged from this review, only one study investigated the presence of 60 PFASs, while all the other studies remained always below the 20 investigated molecules. Despite efforts to reduce the PFASs use in industrial production, their ability to be persistent and bioaccumulative makes them able to continue to impact the food chain. Consequently, monitoring the dietary exposure of humans to a wide range of PFASs is crucial for assessing their effects on human health [46].

However, the diversity in analytical methods—particularly in the choice between traditional SPE and modified QuEChERS—likely contributes to the discrepancies observed in PFAS detection rates across studies. Harmonizing detection limits and validation parameters would significantly improve cross-study comparability.

### 4.3. PFAS Contamination in Non-Cow Milk Species

According to the detection of PFAS in different types of milk, only one study from Poland [35], deepened the topic. This study provides one of the most comprehensive comparisons of PFAS contamination across cow, goat, and sheep milk to date. It highlights significantly higher PFAS levels in goat and sheep milk, suggesting species-specific exposure pathways likely linked to grazing behavior and soil ingestion. Despite low overall dietary risk, these findings underscore the need to deepen research into PFAS contamination beyond cow milk, particularly in small-scale and pasture-based systems that are underrepresented in current literature. This is a significant finding because sheep milk plays a significant role in the dairy economies of several regions, particularly in Asia and Europe. In 2012, global sheep milk production was estimated at approximately 10.12 million tonnes, with Asia contributing the largest share (46.7%), followed by Europe (29.8%) and Africa (23.1%), while the Americas and Oceania accounted for only 0.4% and a negligible amount, respectively [46]. The leading producers globally were China (1.58 million tonnes), Turkey (1.01 million tonnes), and Syria (703,000 tonnes). In Europe, Greece led with 945,430 tonnes, followed by Romania (650,912 tonnes), Spain (552,517 tonnes), Italy (481,970 tonnes), and France (274,686 tonnes). Greece also exhibited the highest per capita consumption of sheep milk, reaching 63.1 kg per year [46,47]. Based on these considerations, this review strongly recommends exploring the topic, especially those countries in which sheep milk is produced and consumed the most.

### 4.4. Risk Characterization

In total, 5 of the 22 studies considered in this review also performed a risk characterization to evaluate human exposure to PFAS through milk consumption. The data presented in this study reflect the year of publication of the individual papers. Consequently, risk characterization assessments and the consequent considerations may not align with the current regulatory limits established by the EFSA.

Xing et al. 2016 [26] conducted a preliminary health risk assessment to evaluate exposure to PFOA and PFOS through milk consumption among the general adult population in Xinjiang, China. The average daily intake of PFOA and PFOS was estimated by combining the mean concentrations of these compounds in milk with daily consumption data obtained from a regional food frequency survey, with results normalized to a standard adult body weight of 60 kg. The study found that PFOS intake (0.0318 ng kg^−1^ per day) was consistently higher than PFOA intake (0.0211 ng kg^−1^ per day) across all population groups. Estimated intakes increased with age, with the highest exposures observed in individuals aged 70 years and above, which correlated with significantly higher milk consumption in this age group (113 g day^−1^). Substantial variability in the average daily intake values was observed across geographic regions, races, and age groups, while gender did not show significant differences. Residents of northern and urban areas, as well as Han Chinese and individuals classified by the Authors as “other races”, probably meaning “other populations”, exhibited the highest intake levels. Despite these variations, the calculated hazard ratios (HRs), based on the very high Tolerable Daily Intakes at that time established by the EFSA (1500 ng kg^−1^ per day for PFOA and 150 ng kg^−1^ per day for PFOS) [15], remained well below 1 for all population subgroups, indicating a negligible health risk from PFAS exposure via milk. The highest observed HR, for PFOS, was 0.362 × 10^−3^. These findings underscore the importance of considering local dietary patterns and demographic factors in PFAS risk assessments.

Macheka et al. 2021 [8] conducted a comprehensive dietary risk assessment to estimate human exposure to PFAS through the intake of dairy milk and infant formula in South Africa, with specific attention to infants, adolescents, and adults. Estimated daily intakes (EDIs) were calculated using age-appropriate milk consumption data from national dietary guidelines and exposure factor handbooks, with separate values derived for urban and rural populations, as well as for partially breastfed and exclusively formula-fed infants. The results revealed that toddlers (ages 1–6) exhibited the highest PFAS intake levels, with total EDIs reaching 20.41 ng kgbw^−1^ per day in urban areas and 14.17 ng kgbw^−1^ per day in rural areas. In comparison, adolescents had the lowest intake levels, and no significant differences were observed between urban and rural populations across age groups, likely due to the similarity in milk sources. Importantly, even in the worst-case scenario—based on WHO-recommended milk consumption volumes—the EDIs for both PFOS and PFOA remained well below the EFSA reference doses (150 ng kgbw^−1^ per day for PFOS and 1500 ng kgbw^−1^ per day for PFOA). However, as previously reported in this review, in 2020 the EFSA [16] established a TWI of 4.4 ng kgbw^−1^, which means that the daily exposure should not exceed 0.6 ng kgbw^−1^. Consequently, these findings do not align with the current regulation. Nonetheless, PFOS levels consistently exceeded those of PFOA across all groups, and toddlers’ overall exposure to PFAS was markedly higher than that of other demographics, underscoring their heightened vulnerability. Particularly notable was the contribution of PFDoA, which accounted for over 30% of the total PFAS intake in all groups, highlighting the need to consider less frequently regulated long-chain PFAS in exposure assessments. While the EDIs available for PFOA and PFOS singularly suggest low immediate health risks, the potential for adverse effects from chronic, cumulative exposure, especially in young children, remains a concern due to the long biological half-lives of PFOS and PFOA (5.4 and 3.8 years, respectively). Moreover, considering the TWI for the sum of the main four PFAS, the results highlight a situation of potential risk. These findings emphasize the importance of incorporating age-specific dietary patterns and cumulative exposure to a broad range of PFAS compounds when assessing the public health risks associated with dairy consumption.

Furthermore, Liu et al. 2022 [34] conducted a human health risk assessment based on hazard quotient (HQ) calculation, which showed that children are significantly more exposed to PFAS through milk consumption than adults, due to higher intake relative to body weight. Using the most recent EFSA reference dose (0.63 ng kgbw^−1^ per day), HQs exceeded 1 in high-exposure scenarios, with values reaching 2.31 for adults and 19.21 for children, indicating a potential health risk even from milk alone. Earlier RfDs resulted in HQs below 1, highlighting how stricter guidelines based on new evidence increase concern. In this study, Monte Carlo simulations confirmed that children consistently fall into higher exposure brackets. These findings emphasize the need to prioritize children in PFAS risk assessments and support stricter regulatory controls on PFAS levels in milk.

According to Mikołajczyk et al. 2023 [35], the intake of PFAS through milk consumption was found to be well below the TWI recommended by EFSA, indicating low health risk. Estimated exposures based on mean upper-bound (UB) concentrations ranged from 0.153–0.266 ng kgbw^−1^ for children and 0.050–0.088 ng kgbw^−1^ for adults, representing less than 7% and 2% of the TWI, respectively. Even under high-exposure scenarios using 95th percentile concentrations and recommended milk consumption levels, intake remained below 48% of the TWI for children and 16% for adults. Cow’s milk, the most commonly consumed, posed no significant concern, with exposure estimates remaining under 20% of the TWI for children even in worst-case scenarios.

Notably, to reach TWI, a child would have to consume over 23 L of milk per week, and an adult over 70 L, which is far beyond typical dietary patterns. However, while milk alone is unlikely to cause an exceedance of the TWI, the cumulative contribution from other PFAS-contaminated foods and water—such as fish or eggs—could lead to higher total exposure. Additionally, the analysis was limited to raw milk; potential PFAS contamination from processing or packaging was not assessed. These findings are consistent with the EFSA conclusion that milk is not a major dietary source of PFAS, though other studies suggest it may contribute significantly to specific contexts.

Xiao et al. 2024 [38] evaluated human health risks from PFAS exposure via milk using EDI and HQ, with a focus on PFOA and PFOS, the most prevalent compounds in milk. The results showed a clear age-related trend, with younger children, especially 1–5 years old, having significantly higher exposure levels than adults due to greater milk intake relative to body weight. Across different regions of China, PFOA and PFOS EDIs decreased with age, and the highest EDIs were observed in 1-year-old children. When applying for the older, more lenient EFSA TDI [14], all HQs remained below 1, indicating no significant health risk. However, with the revised EFSA thresholds from 2018 (0.8 ng kg^−1^ per day for PFOA and 1.8 ng kg^−1^ per day for PFOS) and 2020 (0.63 ng kg^−1^ per day for both), HQs for young children exceeded 1 in most regions, particularly in Central and North China, signaling a potential health risk. For instance, the HQ for 1-year-olds in Central China reached 8.55 for PFOA and 2.99 for PFOS under the 2020 guideline. These findings highlight that children are a vulnerable subgroup in PFAS exposure through milk, underscoring the need for stricter regulatory controls, age-specific risk evaluations, and possibly lower intake recommendations for contaminated milk sources. Although milk alone may not exceed tolerable intake values for the general population, cumulative PFAS exposure from other food and environmental sources should be considered in comprehensive risk assessments.

As an overall consideration, this review confirms that PFASs are detected in milk worldwide and given the variability in the detected concentration levels, it evidences that further research is needed to gather comprehensive data from different regions. Moreover, it would be desirable to define LOQ and LOD values to be mandatory achieved in the various studies to make it possible to compare the results and the detection frequencies more accurately.

Considering the number of analyzed samples, while for some countries such as Italy, Germany, and China, a fair number of samples have been analyzed over the past decade, for many other countries there is little or no data reporting the level of PFAS concentrations in milk. However, even for the nation with a higher number of analyzed samples, the detected PFAS concentrations might be considered indicative, but not representative of the national average concentration. Moreover, a considerable number of samples reported in this review were investigated as they were used as “real samples” in the applications of developed methods. This emphasizes even more the need to set up real monitoring systems on a national scale that might allow us to obtain a true picture of what the actual contamination levels of this matrix might be.

In particular, as emerged from the discussion, PFOS and PFOA are both the most relevant and among the most frequently detected PFAS compounds in the food chain, with PFOS typically having higher detection frequency and mean concentration levels than PFOA. This might be due to the higher bioaccumulation potential of PFOS [24]. Even considering the low concentrations level found in milk for these compounds, the concern is linked to the fact that they have been recently categorized as “carcinogenic to humans” (PFOA) and as “possibly carcinogenic to humans” (PFOS) by the IARC [11].

Therefore, while PFAS levels in milk are generally low and often below the limit of quantification and no significant risk of exposure to PFAS related to milk consumption emerged from the considered studies, milk remains an important route of exposure due to its high consumption deepened. In fact, milk is a fundamental daily food for both adults and, particularly, children [23]. Moreover, while several studies have reported on the concentrations of perfluoroalkyl substances in milk and milk products, only a few have analyzed a wide range of samples and PFAS compounds at the same time. Therefore, while contamination is usually minimal, ongoing vigilance is necessary to manage potential risks to human health [37].

### 4.5. Future Perspectives

The present review highlighted several critical research gaps. Firstly, the scientific literature reveals a clear underrepresentation of studies focusing on non-cow milk. This significantly limits the ability to conduct accurate exposure assessments for populations that regularly or frequently consume these alternative milk types. However, even within the context of cow milk, which is the most studied matrix, the available data remains limited in scope, geographic coverage, and methodological consistency. This overall scarcity of robust and comparable data across different milk types underscores the urgent need for more comprehensive and standardized research efforts to support effective risk assessment and public health decision-making. Secondly, it appears clear that there is a limited temporal coverage of the studies, most datasets reflect short-term or single-timepoint monitoring, which is insufficient to assess seasonal or long-term trends in PFAS accumulation. Research has documented seasonal fluctuations in PFAS concentrations in various environmental media. For instance, a study conducted in the Ebro Delta (Catalonia, Spain) observed that PFOS concentrations in river water varied seasonally, with maximum levels recorded in winter and spring [48]. Research on river waters in central Italy found that the sum of 21 target perfluorinated compounds (PFCs) was highest in June, potentially due to reduced river streamflow during warmer summer months [49]. Despite the documented seasonal variations of PFAS in environmental matrices, there is a notable absence of studies examining the seasonality of PFAS concentrations in cow milk. This gap in the literature highlights the need for targeted research to understand how seasonal factors may influence PFAS levels in milk, which is crucial for accurate exposure assessments and public health evaluations. Eventually, while some studies [4,34] linked PFAS occurrence to agricultural inputs or geographic proximity to pollution sources, systematic source apportionment remains underdeveloped. Future studies and research might start from these considerations and try to explore this uncovered aspect of the topic. Current evidence suggests that fat content and milk processing may influence PFAS distribution in dairy products, although findings are not always statistically significant. For instance, Macheka et al. [8] observed higher PFAS concentrations in full-cream milk, but no significant differences across milk types were confirmed. In contrast, Draghi et al. 2023 [36] reported significantly elevated PFOS levels in milk cream, indicating a potential role of milk processing in modulating PFAS exposure. The same study also found higher detection frequencies in multiparous cows, highlighting a need to explore physiological and dietary factors affecting PFAS bioaccumulation.

Additionally, differences in PFAS levels across animal species and milk types (e.g., cow, sheep, goose), as shown by Mikołajczyk et al. 2023 [35] underscore the importance of understanding the mechanistic basis of PFAS transfer into milk, particularly for long-chain compounds. These findings collectively point to the need for expanded surveillance, toxicokinetic studies, and risk assessments that consider dairy fat content, animal physiology, and milk processing in both contaminated and non-contaminated environments.

As a technical consideration, a valuable perspective for future investigation involves systematically examining how the choice of analytical methods influences PFAS detection in milk, particularly regarding sensitivity and inter-study comparability. Differences in sample preparation techniques—such as solid-phase extraction (SPE) versus modified QuEChERS protocols—and variability in LC-MS/MS instrumentation and settings can significantly affect detection limits and compound recovery. Standardizing analytical workflows would enhance the reliability of cross-study comparisons and support the development of harmonized guidelines for PFAS surveillance in these matrices.

## 5. Conclusions

PFASs have been shown to be ubiquitously present in milk worldwide, with concentrations varying across different countries. The detection of these compounds is usually conducted using high-performance liquid chromatography-tandem mass spectrometry (HPLC-MS/MS), typically involving liquid extraction followed by solid-phase extraction (SPE). However, rapid methods such as QuEChERS have been used to improve the efficiency of the extraction and cleanup steps.

This review highlighted that PFAS contamination in milk has been reported across several geographical areas, including Europe, Asia, Africa, and America. Considering the various PFASs, PFOS and PFOA, reported by the IARC as “carcinogenic to humans” (PFOA) and as “possibly carcinogenic to humans” (PFOS), are among the most frequently detected.

Although the studies reported in the present review showed generally low concentrations of PFASs in milk, it is important to remain aware of potential risks associated with long-term exposure, especially for sensitive population groups (e.g., children and toddlers). Milk, as a widely consumed food item, continues to be an important potential source of PFAS exposure, and consequently ongoing monitoring and updated regulation appears to be crucial to protect human health. This review underlines that further research is essential to assess the impact of these substances on human health and to ensure that appropriate measures are taken to reduce human exposure to PFASs through food, particularly milk.

## Figures and Tables

**Table 1:** Papers published about the detection of PFASs in milk samples in the period between 2014 and 2025. The table shows the investigated analytes, the extraction protocol, and the instrumental technique used to perform the analyses.

Reference	Year	Analytes	Extraction Protocol	Instrumental Analysis
[23]	2014	PFOA; PFOS	L-L extraction followed by two purification steps performed by SPE cartridges	UPLC-MS/MS
[24]	2014	PFBA, PFBS, PFPeA, PFHxA, PFHxS, PFHpA, FHEA, PFOA, PFOS, PFOSA, FOEA, PFNA, PFDA, PFDS, PFUdA, PFDoA, FDEA, PFTrA, PFTeDA, PFHxDA, PFODA	Alkaline digestion followed by a purification step performed by turbulent flow chromatography	LC-QqQ-MS
[25]	2015	PFBA, PFPA, PFHxA, PFHpA, PFOA, PFNA, PFDA, PFUnDA, PFDoDA, PFTrDa, PFTeDA, PFHxDA, PFOcDA, PFBS, PFPS, PFPS, L-PFHxS, L-PFHpS, L-PFOS, L-PFNS, L-PFDS	Extraction and clean up performed using a QuEChERS method	LC-MS/MS
[26]	2016	PFOA; PFOS	Ultrasonic extraction followed by a purification step performed by SPE cartridges	HPLC-MS/MS
[27]	2018	PFHxA, PFOA, PFDA, PFUnDA, PFDoDA, PFHxS, PFOS	Acidification and then purification by SPE cartridges	UPLC-MS/MS
[28]	2018	PFOA, PFOS	Liquid extraction	LC-MS/M
[29]	2018	PFOA, HFPO-DA	Liquid extraction followed by a purification step performed by SPE cartridges	LC-MS/MS
[30]	2020	L- and br-PFOS, PFOA, PFHxS	Alkaline digestion followed by SPE clean-up steps	LC-MS/MS
[8]	2021	PFBA, PFPeA, PFBS, PFHxA, PFHpA, PFHxS, PFOA, PFNA, PFOS, PFDA, PFUdA, PFDS, PFDoA, PFTrDA, PFTeDA	Extraction and clean up performed using a QuEChERS method	UHPLC-MS/MS
[31]	2021	L-PFBS, PFBA, PFDA, PFDoA, PFDS, PFHpA, PFHxA, PFHxS, PFNA, PFOA, PFOS, PFPeA, PFTeDA, PFTrDA, PFuDA	Extraction and clean up performed using a QuEChERS method	UHPLC-MS/MS
[4]	2022	PFBA, PFPeA PFHxA, PFHpA PFOA, PFNA PFDA, PFUnDA PFDoDA, PFTriDA PFTeDA, PFBS, PFPeS, PFHxS PFHpS, PFOS PFNS, PFDS, PFECHS, FBSA FOSA, N-MeFOSA N-EtFOSA, N-MeFOSAA, N-EtFOSAA, 4:2 FtS, 6:2 FtS, 8:2 FtS	Liquid extraction followed by SPE clean up step	LC-MS/MS
[32]	2022	PFBA, PFPeA, PFHxA, PFHpA, PFOA, PFNA, PFDA, PFUnA, PFDoA, PFBS, PFHxS, PFOS, GenX, C6O4	Extraction and clean up performed using a QuEChERS method	LC-MS/MS
[33]	2022	PFPeA, PFHpA PFOA, PFNA PFDA, PFHxS PFOS	Direct immersion SPME (DI-SPME)	LC-MS/MS
[34]	2022	PFBA, PFBS, PFPeA, PFHxA, PFHxS, PFHpA, PFOA, PFOS, PFNA, PFDA, PFUnDA, PFDoDA, PFTrDA, PFTeDA, PFHxDA, PFODA	Liquid extraction followed by SPE clean-up step	UPLC-MS/MS
[35]	2023	PFBS, PFPeS, PFHxS, PFHpS, l-PFOS and br-PFOS, PFHxA, PFHpA, PFOA, PFNA, PFDA, PFUnDA, PFDoA, PFTrDA, PFTeDA	Liquid extraction followed by SPE clean-up step	LC-MS/MS
[36]	2023	PFBA, PFPeA, PFHxA, PFHpA, PFOA, PFNA, PFDA, FOUEA, NADONA, PFBS, PFHxS, PFOS, NmetFOSAA, 6-2FTS	Liquid extraction followed by SPE clean-up step	UPLC-HRMS
[37]	2024	PFPrS, PFBS, PFPeS, PFHxS, PFHpS, PFOS, PFNS, PFDS, PFDoS, PFBA, PFPeA, PFHxA, PFHpA, PFOA, PFNA, PFDA, PFUnDA, PFDoDA, PFTrDA, PFTeDA, PFHxDA, PFOcDA, 4:2FTS, 6:2FTS, 8:2FTS, 10:2FTS, PFEESA, PFECHS, Cl-PFOS, 6:2ClPFESA, 8:2ClPFESA, 3:3FTCA, 5:3FTCA, 7:3FTCA, 8:3FTCA, 6:2FTCA, 8:2FTCA, 10:2 FTCA, 6:2FTUCA, 8:2FTUCA, 10:2FTUCA, PF4OPeA, PF5OHxA, 7H-PFHpA, 11H-PFUnDA, NaDONA, FBSA, FHxSA, FOSA, FDSA, N-MeFBSA, N-MeFOSA, N-EtFOSA, N-MeFOSAA, N-EtFOSAA, N-AP-FHxSA, N-CMAmP 6:2FOSA N-TAmP-FHxSA, 5:1:2FTB, 5:3FTB	pH-dependent cold-induced liquid–liquid extraction as pre-clean-up step	HPLC-MS/MS
[38]	2024	PFBA, PFPeA PFHxA, PFHpA PFOA, PFNA PFDA, PFUnDA PFDoDA, PFTrDA, PFTeDA, PFBS, PFPeS, PFHxS PFHpS, PFOS	Liquid extraction followed by SPE clean-up step	HPLC-MS/MS
[39]	2024	PFBA, PFPeA PFHxA, PFHpA PFOA, PFNA PFDA, PFUnDA PFDoDA, PFTrDA PFTeDA, PFBS PFPeS, PFHxS PFHpS, PFOS PFNS, PFDS PFUnDS, PFDoDS PFTrDS, HFPO-DA DONA	QuEChERS method followed by two clean up steps performed by SPE	LC-HRMS
[40]	2024	PFBA, PFHxA, PFHpA PFOA, PFNA PFDA, PFUnDA PFDoDA, PFTrDA PFTeDA, N-MeFOSAA, N-EtFOSAA, PFBS PFHxS, PFHpS PFOS, PFDS 4:2 FTS, 6:2 FTS 8:2 FTS, L-PENS L-PFDoS	Extraction and clean up performed using a QuEChERS method	LC–MS/MS
[41]	2024	PFBS, PFHxA PFPeS, PFHpA, PFHxS, PFOA, PFHpS, PFNA PFOS, PFDA, PFNS, PFUnDA, PFDS, PFDoDA, PFTrDA, PFDoDS, PFTeA, 4:2 FTS, 6:2 FTS, 8:2 FTS, NaDONA, HFPO-DA	Liquid extraction followed by SPE clean-up step	HPLC-MS/MS
[42]	2025	PFBA, PFPeA PFHxA, PFHpA PFOA, PFNA PFDA, PFUnDA PFDoA, PFTrDA PFTeA, ADONA HFPO-DA (GenX) PFBS, PFPeS PFHxS, PFHpS PFOS, PFNS PFDS, PFDoS 4:2 FTS, 6:2 FTS 8:2 FTS	Liquid extraction followed by a QuEChERS step and then two purification steps performed by SPE	LC-MS/MS

L-L: liquid–liquid; SPE: solid phase extraction; QuEChERS: quick, easy, cheap, effective, rugged and safe; UPLC or UHPLC-MS/MS: ultra-performance liquid chromatography-tandem mass spectrometry; LC-QqQ-MS: liquid chromatography with triple quadrupole mass spectrometry; LC-MS/MS: liquid chromatography-tandem mass spectrometry; HPLC-MS/MS: high-performance liquid chromatography-tandem mass spectrometry.

**Table 2:** Investigated analytes and their frequency of inclusion in the studies considered in this review. The table shows the analytes investigated in at least two different studies.

Analytes	Frequency (%)
PFOA	100
PFOS	100
PFHxS	77
PFHpA	77
PFDA	77
PFHxA	73
PFNA	73
PFBS	64
PFBA	59
PFPeA	55
PFUnDA	55
PFTrDA	50
PFTeDA	50
PFDS	41
PFDoDA	41
PFHpS	36
PFPeS	32
PFDoA	27
6:2 FtS	27
l PFOS	23
br PFOS	23
PFNS	23
HFPO-DA (Gen X)	23
4:2 FtS	23
8:2 FtS	23
PFHxDA	18
NaDONA	14
N-MeFOSAA	14
N-EtFOSAA	14
PFUdA	14
PFDoS	9
PFOcDA	9
PFECHS	9
FBSA	9
FOSA	9
N-MeFOSA	9
N-EtFOSA	9
PFODA	9
PFPS	9
PFTeA	9
PFDoDS	9

## Data Availability

Data is contained within the article or Supplementary Materials.

## Supplementary Materials

The following supporting information can be downloaded at: https://www.mdpi.com/article/10.3390/foods14132274/s1, Table S1: Concentrations of the four main PFASs (PFOA, PFOS, PFNA, PFHxS) detected in milk samples reported by the scientific literature in the period 2014–2025. The results are shown as reported in the original article, without changing the measurement units. If reported in the original article, both the range and the mean/median values are shown.

## Abbreviations

The following abbreviations are used in this manuscript:

10:2 FTCA	10:2 Fluorotelomer carboxylic acid
10:2 FTUCA	10:2 Fluorotelomer unsaturated carboxylic acid
11H-PFUnDA	11H-Perfluoroundecanoic acid
3:3 FTCA	3:3 Fluorotelomer carboxylic acid
5:1:2 FTB	5:1:2 Fluorotelomer betaine
5:3 FTB	5:3 Fluorotelomer betaine
5:3 FTCA	5:3 Fluorotelomer carboxylic acid
6:2 Cl PFESA	6:2 chlorinated polyfluoroalkyl ether sulfonate
6:2 FTCA	6:2 fluorotelomer carboxylic acid
6:2 FTUCA	6:2 Fluorotelomer unsaturated carboxylic acid
7:3 FTCA	7:3 Fluorotelomer carboxylic acid
7H-PFHpA	7H Perfluoroheptanoic acid
8:2 ClPFESA	8:2 chlorinated polyfluoroalkyl ether sulfonate
8:2 FTCA	8:2 Fluorotelomer carboxylic acid
8:2 FTUCA	8:2 Fluorotelomer unsaturated carboxylic acid
8:3 FTCA	8:3 Fluorotelomer carboxylic acid
Cl-PFOS	Chlorinated perfluorooctanesulfonate
FBSA	Perfluoro-1-butanesulfonamide
FDSA	Perfluorodecanesulfonamide
FHxSA	Perfluoro-1-hexanesulfonamide
FOSA	Perfluorooctane sulfonamide
l-PFHxS	Perfluorohexanesulfonic acid
NaDONA	Sodium dodecafluoro-3H-4,8-dioxanonanoate
N-AP-FHxSA	N-(3-dimethylaminopropan-1-yl)perfluoro-1-hexane-sulfonamide
N-CMAmP 6:2FOSA	N-(carboxymethyl)-N,N-dimethyl-N-[3-(1H,1H,2H,2H- perfluoro-1-octanesulfonamido)propan-1-yl]ammonium (6:2 FTAB)
N-TAmP-FHxSA	N-[3-(perfluoro-1-hexanesulfonamido)propan-1-yl]-N,N,N-trimethylammonium
N-EtFOSA	N-ethyl perfluorooctane sulfonamide
N-EtFOSAA	N-ethyl perfluorooctanesulfonamido acetic acid
N-MeFBSA	N-Methylperfluoro-1-butanesulfonamide
N-MeFOSA	N-methyl perfluorooctane sulfonamide
N-MeFOSAA	N-methyl perfluorooctanesulfonamido acetic acid
PF4OPeA	Perfluoro-4-oxapentanoic acid
PF5OHxA	Perfluoro-5-oxahexanoic acid
PFBA	Perfluorobutanoic acid
PFBS	Perfluorobutanesulfonic acid
PFDA	Perfluorodecanoic acid
PFDoDA	Perfluorododecanoic Acid
PFDoS	Perfluorodecane Sulfonic Acid
PFDS	Perfluorodecanesulfonic Acid
PFECHS	Perfluoro-4-ethylcyclohexane
PFEESA	Perfluoro(2-ethoxyethane)sulfonic acid
PFHpA	Perfluoroheptanoic acid
PFHpS	Perfluoroheptanesulfonic acid
PFHxA	Perfluorohexanoic acid
PFHxDA	Perfluoro-n-hexadecanoic acid
PFHxS	Linear perfluorohexanesulfonic acid
PFNA	Perfluorononanoic acid
PFNS	Perfluorononanesulfonic acid
PFOA	Perfluorooctanoic acid
PFOcDA	Perfluorooctadecanoic acid
PFOS	Perfluorooctanesulfonic acid
PFPeA	Perfluoropentanoic acid
PFPeS	Perfluoropentanesulfonic acid
PFTeDA	Perfluorotetradecanoic acid
PFTrDA	Perfluorotridecanoic Acid
PFUnDA	Perfluoroundecanoic acid
10:2 FTS	1H,1H,2H,2H-perfluorododecane sulfonate
4:2 FTS	1H,1H,2H,2H-perfluoro-1-hexanesulfonate
6:2 FTS	1H,1H,2H,2H-perfluoro-1-octanesulfonate
8:2 FTS	1H,1H,2H,2H-perfluoro-1-decanesulfonate
ADONA	Dodecafluoro-3H-4,8-dioxanonanoate
br PFOS	Branched Perfluorooctanesulfonic acid
C6O4	Perfluoropolyether carboxylic acid
FDEA	2-Perfluorodecyl ethanoic acid
FHEA	2-Perfluorohexyl ethanoic acid
FOEA	2-Perfluorooctyl ethanoic acid
FOUEA	2H-perfluoro-2-decenoic acid
HFPO-DA (Gen X)	Hexafluoropropylene oxide dimer acid
l PFDOS	Linear Perfluorodecane Sulfonic Acid
l PFHpS	Linear Perfluoroheptanesulfonic acid
l PFNS	Linear Perfluorononanesulfonic acid
l PFOS	Linear Perfluorooctanesulfonic acid
l-PFBS	Linear Perfluorobutanesulfonic acid
l-PFDS	Linear Perfluorodecanesulfonic Acid
PFDoA	Perfluorododecanoic acid
PFDoDS	Perfluoro-1-dodecanesulfonate
PFODA	4,4′-((perfluorocyclobutane-1,2-diyl)bis(oxy))dianiline
PFOSA	Perfluorooctane sulfonamide
PFPA	Pentafluoropropionic anhydride
PFPrS	Perfluoropropanesulfonic acid
PFTeA	Perfluorotetradecanoic acid
PFTrA	Perfluorotridecanoic acid
PFTrDS	Perfluoro-1-tridecanesulfonate
PFTriDA	Pentacosafluorotridecanoic acid
PFUdA	Perfluoroundecanoic acid
PFUnA	Perfluoroundecanoic acid
PFUnDS	Perfluoroundecane sulfonate
